# Reviewer acknowledgement 2014

**DOI:** 10.1186/s12864-015-1304-z

**Published:** 2015-03-19

**Authors:** Derek E Anane

**Affiliations:** BioMed Central, 236 Gray’s Inn Road, London, WC1X 8HB UK

## Abstract

The editors of BMC Genomics would like to thank all our reviewers who have contributed to the journal in Volume 14 (2014).

**Hiromitsu**

Aaraki Japan

**Federico Abascal**

Spain

**Sophie Abby**

France

**Jason Abernathy**

USA

**Nilwala Abeysekara**

USA

**Moshood Abiola**

Nigeria

**Raed Abu Dawud**

Germany

**Guillaume Achaz**

France

**Luis Actis**

USA

**Bronwen Adams**

UK

**Charles Addo**-**Quaye**

USA

**Zach Adelman**

USA

**David Adelson**

Australia

**Evelien Adriaenssens**

South Africa

**Stein Aerts**

Belgium

**Lydia Afman**

Netherlands

**Suneet Agarwal**

USA

**Ingi Agnarsson**

USA

**Alexander Agoulnik**

USA

**Omar Mario Aguilar**

Argentina

**Gabriela Aguileta**

France

**Zhao Aichun**

China

**Vassilis Aidinis**

Greece

**Elizabeth Ainsworth**

USA

**Eduard Akhunov**

USA

**Fiammetta Alagna**

Italy

**Rob Alba**

USA

**Frank Albert**

USA

**Réka Albert**

USA

**Gabriela Alexe**

USA

**Andrey Alexeyenko**

Sweden

**Tyler Alioto**

Spain

**Andrew Allen**

USA

**Jonathan Allen**

USA

**Jonas Almeida**

USA

**Yannis Almirantis**

Greece

**Enriqueta Alos**

Spain

**Sam Alsford**

UK

**Lucia Altucci**

Italy

**Juan Alvarez**-**Dominguez**

USA

**Rui Alves**

Spain

**Nelson Alves Junior**

Brazil

**Laurence Amar**

France

**Marcel Amichot**

France

**Marcel Amills**

Spain

**Fabian Amman**

Germany

**William Amos**

UK

**Anna Amtmann**

UK

**Yong**-**Qiang An**

USA

**Gema Ancillo**

Spain

**Mark Andersen**

New Zealand

**Kirk Anderson**

USA

**Bjorn Andersson**

Sweden

**Leif Andersson**

Sweden

**Leonardo Andrade**

Brazil

**Jason Andras**

USA

**Erik Andreasson**

Sweden

**Simon Andrews**

UK

**Jean**-**Michel Ane**

USA

**Elisa Angelini**

Italy

**Helal Ansari**

New Zealand

**Juliana Ansari**

USA

**Aldwin Anterola**

USA

**Kristian Apel**

Germany

**Miguel Aranda**

Spain

**Manuel Aranda Lastra**

Saudi Arabia

**Maria Jose Aranzana**

Spain

**Hamutal Arbel**

USA

**Vicent Arbona**

Spain

**David Archer**

UK

**Alan Archibald**

UK

**Danny Arends**

Germany

**Juan Lucas Argueso**

USA

**David Argyle**

UK

**Joaquin Arino**

Spain

**Dan Arking**

USA

**Peter Armbruster**

USA

**John Armour**

UK

**Irena Artamonova**

Russian Federation

**Shuichi Asakawa**

Japan

**Sassan Asgari**

Australia

**Philip Ashton**

UK

**Fred Asiegbu**

Finland

**Maria Asins**

Spain

**Yan Asmann**

USA

**Ulus Atasoy**

USA

**Michael Atchison**

USA

**Peter Atkinson**

USA

**Marcella Attimonelli**

Italy

**Scott Auerbach**

USA

**Brian Austin**

UK

**Simon Avery**

UK

**Gordon Awandare**

Ghana

**Michael Axtell**

USA

**Ferhat Ay**

USA

**Katie Ayers**

Australia

**Nadia Ayoub**

USA

**Rajeev Azad**

USA

**Tatyana Azhikina**

Russian Federation

**Asfar Azmi**

USA

**Ana Paula Moreira**

Brazil

**Igor Babiak**

Norway

**Giridhar Babu**

Korea, South

**Tsvetan Bachvaroff**

USA

**Niclas Backström**

Sweden

**Angshuman Bagchi**

India

**Alessandro Bagnato**

Italy

**Harsh Bais**

Sudan

**Harsh Bais**

USA

**Vladimir Bajic**

Saudi Arabia

**Soren Bak**

Denmark

**Michael Baker**

USA

**Michelle Baker**

Australia

**Richard Baker**

USA

**Rama Balakrishnan**

USA

**Vicente Balanzà**

Spain

**Regina Baldini**

Brazil

**Angela Baldo**

USA

**Eldon Ball**

Australia

**Ana**-**Rosa Ballester**

Spain

**Cengiz Baloglu**

Turkey

**Amitabha Bandyopadhyay**

India

**Anjan Banerjee**

India

**Hua Bao**

Canada

**Weidong Bao**

USA

**Zhilong Bao**

USA

**Fernando Baquero**

Spain

**Abdelali Barakat**

USA

**Pavel Baranov**

Ireland

**Matthew Baranski**

Norway

**Sergio Baranzini**

USA

**Danny Barash**

Israel

**William Barendse**

Australia

**Luca Bargelloni**

Italy

**Guy Barker**

UK

**Jean**-**Francois Baroiller**

France

**Michael Baron**

UK

**Younas Barozai**

Pakistan

**Rodolphe Barrangou**

USA

**Joel Barratt**

Australia

**Cornelius Barry**

USA

**Paul Bartell**

USA

**Afonso Barth**

Brazil

**Jerome Bartholome**

France

**Surajit Basak**

India

**Carole Bassett**

USA

**Filippo Bassi**

Italy

**Nahla Bassil**

USA

**Giorgia Batelli**

Italy

**A K M Abdul Baten**

Australia

**Philip Bates**

USA

**Patrick Bateson**

UK

**Jacqueline Batley**

Australia

**Cristina Battaglia**

Italy

**Juri Battilana**

Italy

**Alexis Battle**

USA

**Carl Bauer**

USA

**Denis Carolin Jessica Bauer**

Australia

**Eva Bauer**

Germany

**Florian F Bauer**

South Africa

**Stefan Bauersachs**

Switzerland

**Jasmin Bavarva**

USA

**Georgii Bazykin**

Russian Federation

**Marco Bazzicalupo**

Italy

**Denise Bazzolli**

Brazil

**Denoyes Béatrice**

France

**Perrin Beatty**

Canada

**Philippe Beaujard**

France

**Andy Beckenbach**

Canada

**Tom Beeckman**

Belgium

**Shaham Beg**

Saudi Arabia

**Luciano Beheregaray**

Australia

**Marcel Behr**

Canada

**Maik Behrens**

Germany

**Susanta Behura**

USA

**Michaël Bekaert**

UK

**Wubishet Abebe Bekele**

Germany

**Eugenio Belda Cuesta**

Spain

**Georgios Belibasakis**

Switzerland

**Terrence Bell**

Canada

**Catherine Bellini**

Sweden

**John Belmont**

USA

**Alexa Bely**

USA

**Marian Bemer**

Netherlands

**Choukri Ben Mamoun**

USA

**Celso Benedetti**

Brazil

**Asa Ben**-**Hur**

USA

**Yuval Benjamini**

USA

**Bernhard Benkel**

Canada

**Chris Benner**

USA

**Panayiotis** (**Takis**) **Benos**

USA

**Touati Benoukraf**

Singapore

**Alfredo Benso**

Italy

**Eric Benson**

USA

**Stephen Bentley**

UK

**Etty** (**Tika**) **Benveniste**

USA

**Gregory Berger**

USA

**Melanie Berkmen**

USA

**Mairead Bermingham**

UK

**Virginie Bernard**

France

**Giorgio Bernardi**

Italy

**Matthias Bernt**

Germany

**David Bertioli**

Brazil

**Edoardo Bertolini**

Italy

**Francesco Bertoni**

Switzerland

**Visnja Besendorfer**

Croatia

**Philippe Bessières**

France

**Venkaiah Betapudi**

USA

**Gerit Bethke**

USA

**Dean Betts**

Canada

**Lothar Beutin**

Germany

**Michael Bevan**

UK

**Andreas Beyer**

Germany

**Luca Bianco**

Italy

**Derek Bickhart**

USA

**Dominique Bicout**

France

**Jean**-**Pierre Bidanel**

France

**Christopher Bidwell**

USA

**Gerd Patrick Bienert**

Germany

**Kyle Biggar**

Canada

**Paul Bilinski**

USA

**James Birchler**

USA

**Terry Bird**

USA

**Nils Birkeland**

Norway

**Inna Biryukova**

Germany

**Alex Bishop**

USA

**Karen Bishop**

New Zealand

**Stephen Bishop**

UK

**Kimberly Bishop**-**Lilly**

USA

**Andrea Bisognin**

Italy

**Douglas Bjelland**

USA

**Pernilla Bjerling**

Sweden

**William Black**

USA

**Guillaume Blanc**

France

**Jorge Blanco**

Spain

**Alexander Blinov**

Russian Federation

**Marc Blondel**

France

**Michael Blower**

USA

**Julien Bobe**

France

**Christoph Bock**

Germany

**Paul Boettcher**

Italy

**Ozren Bogdanovic**

Australia

**Didier Boichard**

France

**Stephane Boissinot**

USA

**Joseph Boland**

USA

**Matei Bolborea**

UK

**Rosa Bolea**

Spain

**Hanna Bolibok**-**Bragoszewska**

Poland

**Aureliano Bombarely**

USA

**Ursula Bond**

Ireland

**Mariangela Bonizzoni**

USA

**Guusje Bonnema**

Netherlands

**David Booth**

Australia

**John Boothroyd**

USA

**Melike Bor**

Turkey

**Anthony Richard Borneman**

Australia

**Tatiana Borodina**

Germany

**Russell Borski**

USA

**Kirill Borziak**

USA

**Jorunn Bos**

UK

**Thomas Bosch**

Germany

**Daniela Bosisio**

Italy

**Emanuela Bostjancic**

Slovenia

**Jose Botella**

Australia

**Miguel Botella**

Spain

**Jean**-**Paul Bouchet**

France

**Pierre Boudry**

France

**Alexandre Bougdour**

France

**Gerrit Bouma**

USA

**Paul Boutros**

Canada

**Henk Bovenhuis**

Netherlands

**Neil Bower**

Australia

**Jon Boyle**

USA

**Kiymet Bozaoglu**

Australia

**Tolga Bozkurt**

UK

**Holger Br&Uuml**;**Ggemann**

Denmark

**Jan Brabek**

Czech Republic

**Martina Bradic**

USA

**Robert Bradley**

USA

**William Bradshaw**

USA

**Siobhan Brady**

USA

**Anja Braeuer**

Germany

**Miguel Branco**

UK

**Rogelio Brandao**

Brazil

**Chris Brandl**

Canada

**Beate Brand**-**Saberi**

Germany

**Kelly Brayton**

USA

**Derrick Brazill**

USA

**Dag Anders Brede**

Norway

**Marcus Breese**

USA

**Haim Breitbart**

Israel

**Bertram Brenig**

Germany

**Christelle Breton**

France

**Jason Breves**

USA

**Tiziana Brevini**

Italy

**Scott Briggs**

USA

**Marcelo Brigido**

Brazil

**Francoise Bringel**

France

**Thorsten Brinkhoff**

Germany

**Fiona Brinkman**

Canada

**Luciano Brocchieri**

USA

**Guy Brock**

USA

**Gudrun Brockmann**

Germany

**Peter Bron**

Netherlands

**John Brookfield**

UK

**Yariv Brotman**

Germany

**Andrew Brown**

UK

**Steven Brown**

USA

**Titus Brown**

USA

**Daniel Brown**

USA

**Paul Brown**

USA

**Patrick Brown**

USA

**Sigal Brown Miyara**

Israel

**Jeremy Bruenn**

USA

**Kevin Bruhn**

USA

**Lone Bruhn Madsen**

Denmark

**Søren Brunak**

Denmark

**Véronique Brunaud**

France

**Frederic Brunet**

France

**Vincent Bruno**

USA

**Katarzyna Bryc**

USA

**Rong Bu**

Saudi Arabia

**Hikmet Budak**

Turkey

**Bruce Budowle**

USA

**Catherine Robin Buell**

USA

**Florian Buettner**

Cote d'Ivoire

**Alfonso Buil**

Switzerland

**Albert Johannes Buitenhuis**

Denmark

**Tim Bull**

UK

**Tom Burdon**

UK

**Colleen Burge**

USA

**Lyle Burgoon**

USA

**Michael Burke**

USA

**Thorsten Burmester**

Germany

**Karen Burnett**

USA

**Alexander Burroughs**

Japan

**Antje Burse**

Germany

**Vincent Bus**

Nicaragua

**Jill Bushakra**

USA

**Pierre Bushel**

USA

**Pietro Buzzini**

Italy

**Zijun Caaslyh**

China

**Armando Caballero**

Spain

**Mark Caddick**

UK

**Raquel Cadete**

Brazil

**Hongmei Cai**

China

**Xuepeng Cai**

China

**James Cai**

USA

**Richard Calendar**

USA

**Seam Callahan**

USA

**Tercilio Calsa Junior**

Brazil

**Juan Calvete**

Spain

**Rory Andrew Cameron**

USA

**Stephen Cameron**

Australia

**Fabien Campagne**

USA

**Michael Campana**

USA

**Stefano Campanaro**

Italy

**Adelino Canario**

Portugal

**Leonor Cancela**

Portugal

**Hector Candela**

Spain

**Sander Canisius**

Netherlands

**Steven Cannon**

USA

**David Canovas**

Spain

**Dario Cantu**

USA

**Chunyang Cao**

China

**Blanche Capel**

USA

**Aurélien Capitan**

France

**Antonio Carapelli**

Italy

**Fabrizio Carbone**

Italy

**Carsten Carlberg**

Finland

**Jane Carlton**

USA

**Jason Carlyon**

USA

**John Carman**

USA

**Piero Carninci**

Japan

**Agamemnon Carpousis**

France

**Mary Carrington**

USA

**Jason Carroll**

UK

**Richard Carter**

UK

**Oliviero Carugo**

Austria

**Fabiola Carvalho**

Brazil

**Michael Carvan**

USA

**Josep Casadesus**

Spain

**Margarida Casal**

Portugal

**Nicola Casali**

UK

**Eduardo Casas**

USA

**Nicholas Casewell**

UK

**Sergi Castellano**

Germany

**Simone Castellarin**

Canada

**Bianca Castiglioni**

Italy

**Raquel Catarino**

Portugal

**Dominique Caugant**

Norway

**Mathilde Causse**

France

**Pablo Federico Cavagnaro**

Argentina

**Andrea Cavallini**

Italy

**Rick Cavicchioli**

Australia

**Luigi Ceci**

Italy

**Jean**-**Marc Celton**

France

**Gustavo Cerqueira**

USA

**Gustavo Cerqueira**

Australia

**Stefano Cesco**

Italy

**David Chagne**

New Zealand

**Richard J Chaillet**

USA

**Parin Chaivisuthangkura**

Thailand

**Jayprokas Chakrabarti**

India

**Sounak Chakraborty**

USA

**Ken Chalmers**

Australia

**Jonathan Chan**

Thailand

**Zhulong Chan**

China

**Joel Chandlee**

USA

**Shih**-**Ching Chang**

Taiwan

**Ming**-**Ling Chang**

Taiwan

**Woo**-**Suk Chang**

USA

**Nathalie Chantret**

France

**Hubert Charles**

France

**Jean**-**Louis Charli**

Mexico

**Gilles Charmet**

France

**Pep Charusanti**

USA

**Christine Chase**

USA

**Raghunath Chatterjee**

India

**Dipankar Chatterji**

India

**Elena Chaves**-**Pozo**

Spain

**Charles Chen**

USA

**Hong Chen**

China

**Jin**-**Gui Chen**

USA

**Rongjun Chen**

China

**Chih**-**Hao Chen**

Taiwan

**Jian Chen**

China

**Qijun Chen**

China

**Fan Chen**

China

**Jianchi Chen**

USA

**Jin Chen**

USA

**Jinfeng Chen**

USA

**Li**-**Song Chen**

China

**Shi Chen**

China

**Shilin Chen**

China

**Wei**-**Jung Chen**

Taiwan

**Yinglong Chen**

Australia

**Yizhou Chen**

Australia

**Yong Chen**

China

**Chao Cheng**

USA

**Han Cheng**

China

**Hans Cheng**

USA

**Yunjiang Cheng**

China

**Ekaterina Chernyaeva**

Russian Federation

**Anne**-**Marie Chevre**

France

**Vikram Chhatre**

USA

**Parveen Chhuneja**

India

**Vincent Chiang**

USA

**Yu**-**Chung Chiang**

Taiwan

**Marcos Chiaratti**

Brazil

**Sabina Chiaretti**

Italy

**Kevin Childs**

USA

**Anong Chirapart**

Thailand

**Ludmila Chistoserdova**

USA

**Joanna Chiu**

USA

**Doil Choi**

Korea, South

**Je**-**Yong Choi**

Korea, South

**Murim Choi**

USA

**Sun Shim Choi**

Korea, South

**Kang Chong**

China

**Hui**-**Hsien Chou**

USA

**Mani Choudhary**

USA

**Valerie Choumet**

France

**Henrik Christensen**

Denmark

**Riann Christian**

South Africa

**Julian Christians**

Canada

**George Christophides**

UK

**Jeffrey Shih Chieh Chu**

Canada

**Ka Hou Chu**

Hong Kong

**Mingxing Chu**

China

**Jeffrey Chuang**

USA

**Dmitriy Chudakov**

Russian Federation

**I**-**Fang Chung**

Taiwan

**Edward Chuong**

USA

**Gennady Churakov**

Germany

**Salvatrice Ciccarese**

Italy

**Francesco Cicconardi**

Italy

**Mete Civelek**

USA

**Marcus Claesson**

Ireland

**Clifford Clark**

Canada

**Leigh Clark**

USA

**Michael Clark**

UK

**Ian Clarke**

UK

**Manuel Gonzalo Claros**

Spain

**Rollie Clem**

USA

**Melissa Cline**

USA

**Beatrice Clouet**-**D**’**Orval**

France

**Steve Clough**

USA

**Jeremy Mark Cock**

France

**Sergio Cocozza**

Italy

